# High Primary Antibiotic Resistance of *Helicobacter pylori* Strains Isolated from Pediatric and Adult Patients in Poland during 2016–2018

**DOI:** 10.3390/antibiotics9050228

**Published:** 2020-05-02

**Authors:** Paweł Krzyżek, Dorota Pawełka, Barbara Iwańczak, Radosław Kempiński, Konrad Leśniakowski, Francis Mégraud, Łukasz Łaczmański, Monika Biernat, Grażyna Gościniak

**Affiliations:** 1 Department of Microbiology, Wroclaw Medical University, 50-368 Wroclaw, Poland; grazyna.gosciniak@umed.wroc.pl; 2 Department and Division of Surgical Didactics, Wroclaw Medical University, 50-368 Wroclaw, Poland; dorotapawelka@wp.pl; 3 Department and Clinic of Pediatrics, Gastroenterology and Nutrition, Wroclaw Medical University, 50-369 Wroclaw, Poland; barbara.iwanczak@umed.wroc.pl; 4 Department of Gastroenterology and Hepatology, Wroclaw Medical University, 50-556 Wroclaw, Poland; radoslaw.kempinski@umed.wroc.pl; 5 J. Gromkowski Regional Specialist Hospital, 51-149 Wroclaw, Poland; konradlesniakowski@op.pl; 6 Centre National de Référence des Campylobacters et Hélicobacters, Université de Bordeaux, 33076 Bordeaux, France; francis.megraud@chu-bordeaux.fr; 7 Hirszfeld Institute of Immunology and Experimental Therapy, Polish Academy of Sciences, 53-114 Wroclaw, Poland; lukasz@diagmol.com; 8 Department of Haematology, Blood Neoplasms, and Bone Marrow Transplantation, Wroclaw Medical University, 50-367 Wroclaw, Poland; mobiernat@gmail.com

**Keywords:** *Helicobacter pylori*, antibiotic resistance, antibiotic susceptibility, clarithromycin, metronidazole, levofloxacin

## Abstract

Monitoring the antibiotic resistance of *H. pylori* is an important step in the effective treatment of this bacterium, thus the aim of the present study was to assess the prevalence of antimicrobial resistance of *H. pylori* strains isolated from pediatric and adult patients with primary infections in 2016–2018. Antral biopsies from 334 treatment-naïve patients (126 children and 208 adults) were obtained. A total of 71 clinical *H. pylori* strains (22 from children and 49 from adults) were isolated and examined for amoxicillin (AMX), clarithromycin (CLR), metronidazole (MTZ), tetracycline (TET), and levofloxacin (LEV) susceptibility. The activity of the antibiotics was measured by E-tests. Strains were considered as resistant to antibiotics with minimum inhibitory concentrations (MICs) equal to ≥0.125 μg/mL (AMX), ≥0.5 μg/mL (CLR), ≥8 μg/mL (MTZ), and ≥1 μg/mL (TET and LEV). The highest prevalence of antibiotic resistance in *H. pylori* strains was observed for CLR and MTZ, at frequencies of 54.5% and 31.8% vs. 30.6% and 46.9% for children and adults, respectively. A much lower frequency of isolation of resistant strains was demonstrated for LEV and TET, this being 9.1% and 4.5% vs. 18.4% and 4.1% for pediatric and adult patients, respectively. The presence of AMX-resistant strains was not observed. The *H. pylori* strains isolated from Polish patients with primary infections showed a high level of antibiotic resistance to CLR and MTZ (>30%).

## 1. Introduction

Infectious diseases have been a challenge for humanity since the dawn of time [1]. Improving sanitation and hygiene conditions has enhanced this situation, while the discovery and widespread introduction of antibiotics is considered a breakthrough moment in the fight against microbes. These compounds revolutionized medicine and significantly improved our quality of life [2]. Their use, however, is not without side effects, with the most important being the massive and progressive spread of antibiotic-resistant microorganisms [1,2,3,4]. The problem of modern antibiotic therapy is associated with the very rare introduction of new substances, with most currently available having been discovered in the mid-20th century [1,2]. To raise public awareness of the threat arising from the growing problem of antibiotic resistance, in 2017 WHO created a list of priority pathogens [5].

Among the catalog of the 12 families/genera of bacteria posing the greatest threat to human health, *Helicobacter pylori* was mentioned [5]. Classically, this bacterium is acquired during childhood and is capable of establishing lifelong colonization [6]. In most cases, such individuals remain asymptomatic, while clinical symptoms may include a wide range of gastropathies, including gastric ulcers and cancers [6,7,8]. Given the high prevalence of *H. pylori* (half of humanity) and the significant impact on health and mortality, proper assessment and effective treatment of infections caused by this bacterium seem to be of immense importance [8]. One of the most important problems in *H. pylori* therapy is the high heterogeneity of this microorganism, resulting from intensive horizontal gene transfer and high mutation frequency [6,7,9,10]. Therefore, constant monitoring of *H. pylori* resistance, especially the primary one indicating patients’ regional antibiotic intake profile, is an important process of limiting the prevalence of this bacterium and reducing its harmful effect on human health [7,8,10,11].

The aim of the present study was to determine the primary antibiotic resistance of *H. pylori* in pediatric and adult patients in Southern Poland in 2016–2018. In addition, a relationship between gender and age and the frequency of *H. pylori* resistance to specific antibiotics was also determined.

## 2. Materials and Methods

**Figure 1:**
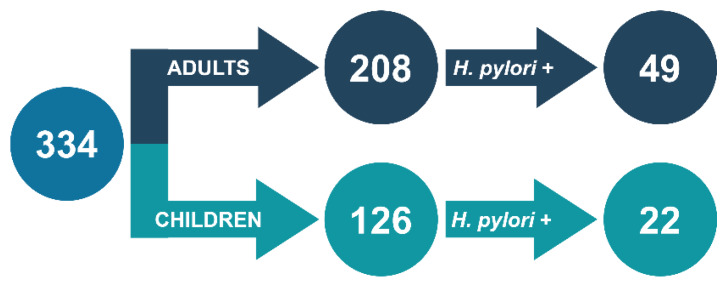
Patient population and amount of *H. pylori* strains included in the study.

*H. pylori* strains were isolated at the Department of Microbiology, Wroclaw Medical University, Poland in 2016–2018 from biopsies obtained during routine gastroscopic examinations in four medical units in Wroclaw, Poland: Department and Clinic of Pediatrics, Gastroenterology and Nutrition; Department and Division of Surgical Didactics; Department of Gastroenterology and Hepatology; and J. Gromkowski Regional Specialist Hospital. Overall, 334 treatment-naïve patients were examined, including 126 children (0–15 years, 55 males and 71 females) and 208 adults (≥16 years, 68 males and 140 females). A total of 71 *H. pylori* strains from 22 pediatric (9 males and 13 females) and 49 adult (17 males and 32 females) patients were grown (Figure 1). Bacterial identification was made by confirming the positive reaction of urease, catalase, and oxidase, and the presence of spiral/curved Gram-negative rods. The research was approved by the bioethics commission of the Wroclaw Medical University (#111/2017 and #203/2019).

Bacterial cultures with a density of 3 McFarland scale units were sown on Mueller–Hinton (Becton Dickinson, Le Pont de Claix, France) media with 5% horse blood and incubated for 3–5 days under microaerophilic conditions and 37 °C. Determination of antibiotic sensitivity was performed using E-tests with amoxicillin (AMX, range: 0.016–256 µg/mL), clarithromycin (CLR, range: 0.016–256 µg/mL), metronidazole (MTZ, range: 0.016–256 µg/mL), tetracycline (TET, range: 0.016–256 µg/mL), and levofloxacin (LEV, range: 0.002–32 µg/mL), all from BioMerieux. *H. pylori* strains were considered resistant with the following limits: ≥0.125 µg/mL (AMX), ≥0.5 µg/mL (CLR), ≥1 µg/mL (TET and LEV) and ≥8 µg/mL (MTZ), in accordance with EUCAST 2019 recommendations [12].

Statistical analysis was performed using the STATISTICA program.

## 3. Results

**Figure 2:**
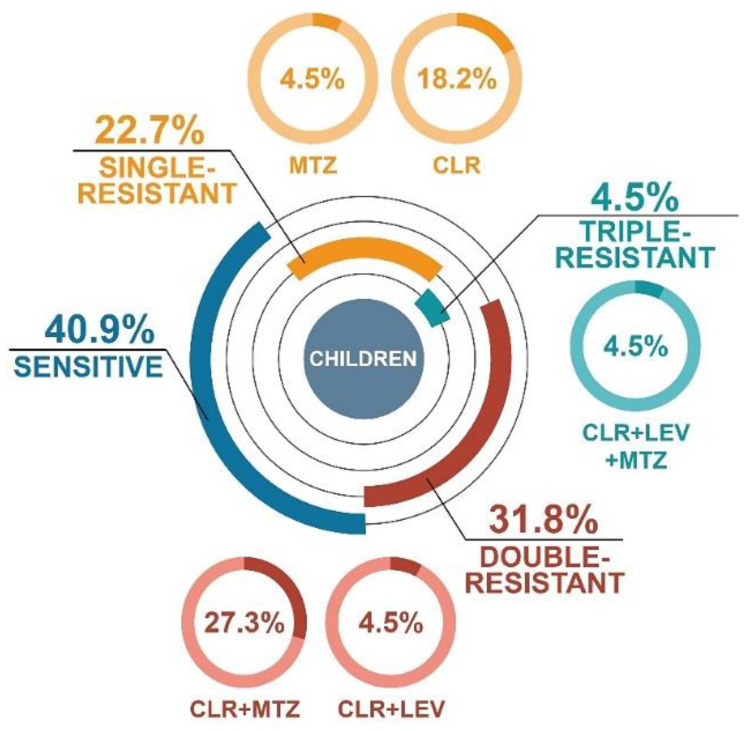
Distribution of resistance profiles of *H. pylori* strains isolated from treatment-naïve pediatric patients from Poland during 2016–2018.

Among *H. pylori* isolates from pediatric patients, less than half of them were sensitive to the antibiotics tested (40.9%) (Figure 2). There were 22.7% single-resistant (18.2% CLR and 4.5% MTZ) and 31.8% double-resistant (27.3% CLR + MTZ and 4.5% CLR + LEV) strains. One isolate was triple-resistant (4.5%, CLR + LEV + TET). It is noteworthy that among thirteen resistant *H. pylori* strains obtained from children, eleven of them had monoresistance or co-resistance to CLR.

**Figure 3:**
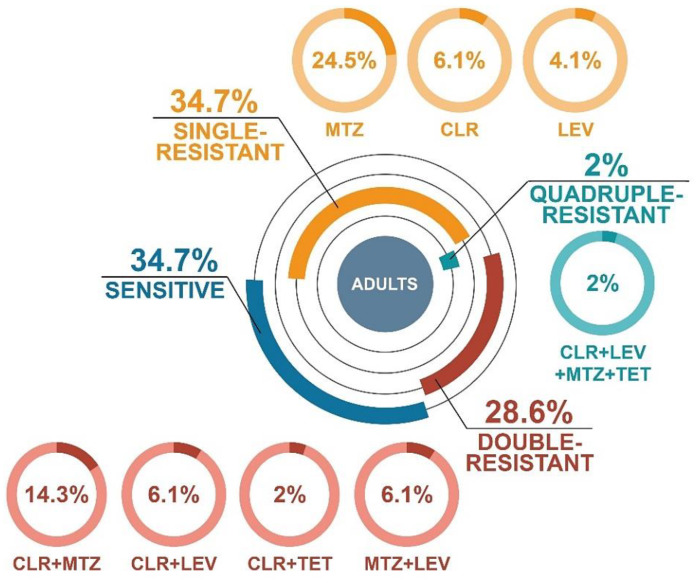
Distribution of resistance profiles of *H. pylori* strains isolated from treatment-naïve adults from Poland during 2016–2018.

The resistance pattern in *H. pylori* isolates grown from adults had a similar distribution as in pediatric patients (Figure 3). In this case, the amount of both sensitive and single-resistant strains was 34.7% each. In the group of *H. pylori* strains with single resistance, MTZ resistance predominated (24.5%). Isolates with double resistance accounted for 28.6% (14.3% CLR + MTZ, 6.1% MTZ + LEV, 6.1% CLR + LEV, and 2% CLR + TET). One strain with quadruple resistance (2%, CLR + MTZ + TET + LEV) was observed.

**Figure 4:**
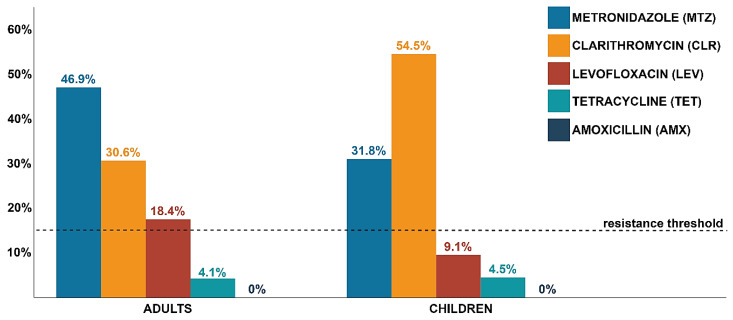
The frequency of resistance to specific antibiotics among *H. pylori* strains isolated from treatment-naïve adult and pediatric patients from Poland during 2016–2018. The resistance threshold is equal to 15% and is considered as a contractual value above which the use of an antibiotic should not be taken into account in empirical therapies [13].

**Figure 5:**
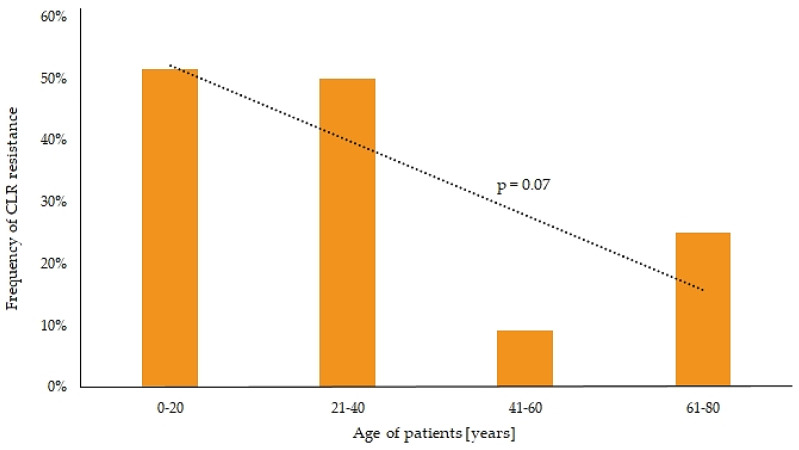
Tendency between the frequency of clarithromycin (CLR) resistance among *H. pylori* strains and the age of patients.

The highest frequencies of resistance among *H. pylori* strains were observed for CLR and MTZ, which were 30.6% and 54.5% and 46.9% and 31.8% in adults and children, respectively (Figure 4). Much lower prevalence was observed for LEV and TET, this being 18.4% and 9.1% and 4.1% and 4.5% in adult and pediatric patients, respectively. No AMX-resistant strains were seen in any group. Only for CLR-resistant *H. pylori* strains a statistically significant difference in the incidence between children and adults (*p* = 0.05) was observed (Figure 4). In addition, there was a negative tendency between increasing age and CLR resistance (*p* = 0.07) (Figure 5). The incidence of CLR-resistant *H. pylori* strains was around 50% in patients aged 0–40 years and it decreased in older age groups (9% and 25% in people aged 41–60 and 61–80 years, respectively). No relationship was seen between gender and resistance to any antibiotic (*p* > 0.05).

## 4. Discussion

The crisis regarding antibiotic resistance is currently one of the most serious problems affecting humanity [3,14]. There are several factors responsible for this phenomenon, e.g., inadequate diagnostics (broad spectrum antibiotics, poorly selected antibiotics, antibiotic therapy for viral and fungal infections), inadequate dosage of drugs, and low vaccination frequency [14]. Analysis of global antibiotic consumption over the years 2000–2015 showed an increase in the use of these substances by 65%, and in the absence of appropriate programs controlling antibiotics consumption, in the next 15 years their intake will increase by 200% [15].

The use of antibiotics exerts selective pressure on microorganisms that colonize the human body [4]. Therefore, patients undergoing antibiotic therapies more often are exposed to a higher risk of infections with microbes resistant to antimicrobial substances. This is particularly important for bacteria colonizing their hosts for a prolonged period of time, such as *H. pylori* [6]. Exposure to monotherapy, aimed at eradicating other microorganisms, does not eliminate infection produced by *H. pylori*. Such treatment, however, can give the "bottleneck effect" on the population of this bacterium, contributing to the development of *H. pylori* populations resistant to specific groups of antibiotics.

The current article shows a high level of primary antibiotic resistance of *H. pylori* strains isolated from adults (30.6%, 46.9% and 18.4% for CLR, MTZ, and LEV, respectively) and children (54.5%, 31.8% and 9.1% for CLR, MTZ, and LEV, respectively). Reviewing reports from the last five years, it has been noticed that this prevalence is consistent with data from many European countries, including France [16], Italy [17,18], Spain [19,20] and Portugal [21], and concurrent with the multicenter study on *H. pylori* primary resistance in Europe from 2013 [11]. Comparison of our results with the study conducted 10 years ago in our research unit (Poland), regarding the primary resistance of *H. pylori*, indicates a persistent high level of CLR and MTZ resistance in both children and adults [22]. However, an increase in LEV resistance over the decade from 1.9% to 9.1% in pediatric patients and from 11.7% to 18.4% in adults has been observed. This is in line with reports from another research center in Poland, indicating an increasing resistance of *H. pylori* strains to LEV [23].

The alarming level of primary resistance of *H. pylori* is most likely correlated with the growing and uncontrolled intake of antibiotics used in the eradication of this bacterium [13]. This is confirmed by the global report on the consumption of antibiotics, which showed that the five most frequently consumed groups of these substances are β-lactams (penicillins and cephalosporins), macrolides, fluoroquinolones, tetracyclines, and trimethoprim [15]. Many studies have recognized the existence of a strong correlation between the intake of fluoroquinolones (urinary and respiratory tract infections), macrolides (respiratory tract infections), and nitroimidazoles (gynecological, parasitological and dental infections) in the treatment of other infections and the development of resistant *H. pylori* strains [11,24,25,26,27]. In the present study, the prevalence of CLR-resistant strains in children (54.5%) was almost twice as high as in adults (30.6%). Such a large discrepancy seems to be caused by a higher susceptibility of pediatric patients to respiratory infections and more frequent consumption of macrolides by this age group [28]. In addition, in the current study, a higher prevalence of LEV- and MTZ-resistant isolates was found in adults (18.4% and 46.9%, respectively) than pediatric patients (9.1% and 31.8%, respectively). These differences, although not statistically significant, correlate with reports of others, indicating a positive relationship between age of >50 years (higher exposure to urogenital infections) and the presence of *H. pylori* strains primarily resistant to LEV and MTZ [11,24,25]. The results obtained in this article seem to confirm the relationship between frequent consumption of antibiotics and the spread of *H. pylori* strains with primary resistance.

With the increase in resistance to antibiotics in *H. pylori* stains, appropriate attention should be paid to the choice of therapy (taking into account the local resistance profile and history of consumed antibiotics) [8]. The biggest challenge in the treatment of *H. pylori* in the world is resistance to CLR and LEV [9]. Resistance to MTZ although wide-spread can be overcome in vivo by extending the duration of therapy (up to 14 days) and increasing the dose (up to 1.5 g/day) [7,9,13]. Since the incidence of primary resistance to CLR and LEV in *H. pylori* strains isolated in Poland has exceeded 15%, the conventional threshold of acceptable prevalence [13], therapies with these antibiotics is discouraged. Based on this, according to the authors of the article, it is reasonable to use bismuth quadruple therapy (bismuth salts, MTZ, TET, and proton pumps inhibitor [PPIs]) as a first line treatment [7,29], because in this case resistance to MTZ does not significantly affect the therapeutic result [30,31]. It should be borne in mind, however, that this applies to strains with low resistance levels (8–32 µg/mL), and higher resistance (>32 µg/mL) may reduce the effectiveness of this therapy from >90% to 60% [32]. Very promising results in the Asian population have been obtained for high-dose double therapy (AMX and PPIs), i.e., eradication rate >90% and low level of side effects (<15%) [33,34]. Avoiding the use of antibiotics with high frequency of resistance among *H. pylori* strains and the use of AMX, to which no resistance is currently observed in Poland, give hope for a wider use of this therapy. Research on the European population is undoubtedly relevant.

## 5. Conclusions

The level of primary resistance to CLR and MTZ of *H. pylori* strains in both adults and children in Poland is high (>30%). The prevalence of CLR resistance is negatively correlated with increasing age and is almost twice as high in pediatric patients as in adults.
